# Single-Cell Sequencing Reveals the Novel Role of Ezh2 in NK Cell Maturation and Function

**DOI:** 10.3389/fimmu.2021.724276

**Published:** 2021-10-26

**Authors:** Minghang Yu, Ziyang Su, Xuefeng Huang, Xi Wang

**Affiliations:** ^1^ Beijing Key Laboratory of Emerging Infectious Diseases, Institute of Infectious Diseases, Beijing Ditan Hospital, Capital Medical University, Beijing, China; ^2^ Department of Immunology, School of Basic Medical Sciences; Advanced Innovation Center for Human Brain Protection, Beijing Key Laboratory for Cancer Invasion and Metastasis, Department of Oncology, Capital Medical University, Beijing, China

**Keywords:** NK cell, Ezh2, maturation trajectory, cytotoxic function, AP-1, scRNA-seq

## Abstract

Natural killer (NK) cells are lymphocytes primarily involved in innate immunity and exhibit important functional properties in antimicrobial and antitumoral responses. Our previous work indicated that the enhancer of zeste homolog 2 (Ezh2) is a negative regulator of early NK cell differentiation and function through trimethylation of histone H3 lysine 27 (H3K27me3). Here, we deleted Ezh2 from immature NK cells and downstream progeny to explore its role in NK cell maturation by single-cell RNA sequencing (scRNA-seq). We identified six distinct NK stages based on the transcriptional signature during NK cell maturation. Conditional deletion of Ezh2 in NK cells resulted in a maturation trajectory toward NK cell arrest in CD11b SP stage 5, which was clustered with genes related to the activating function of NK cells. Mechanistically, we speculated that Ezh2 plays a critical role in NK development by activating AP-1 family gene expression independent of PRC2 function. Our results implied a novel role for the Ezh2-AP-1-Klrg1 axis in altering the NK cell maturation trajectory and NK cell-mediated cytotoxicity.

## Introduction

Natural killer (NK) cells are lymphocytes belonging to innate immunity with effector functions, including roles as cytolytic effectors and potent producers of cytokines (1–3). In the bone marrow (BM) of mice, the NK cell lineage derives from common lymphoid progenitors (CLPs) through the acquisition of CD122 expression (4). After acquiring NK1.1 and CD49b surface expression, murine NK cells can further develop to maturity (4, 5). NK cell maturation *in vivo* follows the pathway CD11b^low^CD27^high^ (CD27 single positive, CD27 SP) → CD11b^high^CD27^high^ (CD11b CD27 double positive, DP) → CD11b^high^CD27^low^ (CD11b single positive, CD11b SP) (6). CD11b^high^ NK cells display potent effector functions compared with CD27^high^ NK cells (6). Klrg1 is a marker of terminal maturation acquired by NK cells (7, 8). Interestingly, Scott *et al.* found that Klrg1 is a C-type lectin-like inhibitory receptor with an immune receptor tyrosine-based inhibitory motif in its cytoplasmic domain during the activation of mouse NK cells (9). Masayuki *et al.* found that *Klrg1* binds to members of the classical cadherin family and inhibits NK cell cytotoxicity (10). The Klrg1^+^ ILC2 pool has recently been shown to be impaired in mice deficient in BATF, a member of the AP-1 superfamily (11), implying a potential role of the AP-1 superfamily in the regulation of the Klrg1^+^ lymphocyte population.

Ezh2 is the catalytic subunit of polycomb repressive complex 2 (PRC2) (12) and has been traditionally known to mediate histone H3K27 trimethylation, a hallmark of silent chromatin (13). However, Ezh2 also promotes gene expression in cancer cells without being dependent on its methyltransferase activity. Ezh2 activates gene expression based on a transactivation domain (TAD) capable of interacting with components of the active transcription machinery (14). Shi B et al. found that Ezh2 physically binds to estrogen receptor α (ERα), β-catenin, and mediators to transactivate promoters of downstream genes in MCF-7 ER-positive breast cancer cells (15). In addition, Jung et al. also showed that the PRC2-independent interaction between Ezh2 and PAF (PCNA-associated factor) mediates activation of β-catenin target genes in colon cancer cells (16). We previously reported that deletion or inhibition of Ezh2 in hematopoietic stem and progenitor cells (HSPCs) enhanced NK cell commitment and cytotoxicity against tumor cells (17). However, whether Ezh2 regulates NK cell development and function at a later stage and how Ezh2 exerts its biological activity remain elusive.

In this study, NK cell maturation was divided into six detailed stages at the transcriptome level from early CD27 SP stage 1 to Klrg1 high stage 6. We further showed that altered NK-mediated cytotoxic function is intrinsically associated with altered developmental processes. Furthermore, *Ezh2^-/-^* NK cells followed a maturation trajectory toward arrest in CD11b SP stage 5. We found that the Klrg1 high stage corresponded to a late population to the CD11b SP stage. *Itgam* is clustered with genes known to enhance NK cell cytotoxic function through a pseudotemporal expression pattern (18–20), while *Klrg1* shows the opposite orientation (21–24). Furthermore, we found that the transcriptional levels of AP-1 family members were consistently downregulated in *Ezh2*-deficient NK cells. Motifs distributed across promoters of genes from the AP-1 superfamily are most similar to the estrogen-related receptor (ERR) family binding site. In conclusion, our results implied a novel role for the Ezh2-AP-1-Klgr1 axis in altering the NK cell maturation trajectory and enhancing NK cell-mediated cytotoxicity.

## Materials And Methods

### Mice

*Ezh2^fl/fl^* mice were purchased from the Jackson Laboratory (Bar Harbor, ME, USA). *Ncr1^iCre^* mice were purchased from Beijing Biocytogen (Beijing, China). To generate Ezh2 deletion in NK cells, *Ezh2^fl/fl^* mice were crossed with *Ncr1^iCre^* mice to obtain *Ezh2^fl/fl^;Ncr1^iCre^* mice (*Ezh2^ΔNK^*). All mice were housed in a specific pathogen-free facility for use according to the guidelines for experimental animals at Capital Medical University. All animals used were C57BL/6 mice aged 6 to 8 weeks.

### NK Cell Separation, Library Construction, and Sequencing

Spleens were mechanically disrupted in PBS, and single-cell suspensions were prepared as described previously (17). For cell sorting, bulk spleen cells were used to isolate CD45^+^CD3^-^CD19^-^NKp46^+^NK1.1^+^ cells for scRNA-seq experiments by the BD FACSAria III instrument, and the purity was generally above 95%. Library construction was performed using a BD Rhapsody™ Single-Cell Analysis System following a standard protocol provided by the manufacturer (BD Biosciences) (25). RNA sequencing was performed on an Illumina HiSeq 2500 machine with a sequencing depth of at least 50,000 reads per cell.

### ScRNA-seq Data Analysis

Sequenced reads were aligned to the GRCm38 murine transcriptome, and then the expression of transcripts in each cell was quantified using the BD™ Rhapsody Whole Transcriptome Assay Analysis Pipeline. Downstream analyses were implemented using R (v4.0.2) and the package iCellR. Low-quality cells were excluded in the initial quality control (QC) step by removing cells with fewer than 500 genes expressed or more than 2,400 genes. We also removed cells with mitochondrial transcript contents greater than 5%. As a result, 2,584 *Ezh2^-/-^* NK cells and 2,612 WT NK cells were retained, and 2,584 WT NK cells were selected to achieve equal group numbers for further analysis. The function norm.date (norm.method = “ranked.glsf”, top.rank = 500) was applied for data normalization.

### Dimension Reduction, Unsupervised Clustering, Cell Type Prediction, and Developmental Trajectory Inference

Variable genes were selected by the make.gene.model function (dispersion.limit = 1.5, base.mean.rank = 500, no.mito.model = T, mark.mito = T, interactive = F). Ribosomal genes were removed. Then, the variable genes were used for PCA implemented with the run.pca function. Next, we selected PCs 1–10 as input for tSNE and UMAP analyses or 1–20 for KnetL analysis. Cells were clustered with iCellR’s iclust function. Marker genes were identified by the findMarkers function (fold.change = 1.5, padjval = 0.1), and then top markers were determined (marker.genes, topde = 10, min.base.mean = 0.05, filt.ambig = F). Cell type prediction was performed by the cell.type.pred function (immgen.data = “uli.rna”, gene = maerker.genes, plot.type = “point.plot”, top.cell.types =10) based on the ImmGen Ultra Low Input (ULI) RNA-seq dataset. Pseudotime was generated with Monocle v2 to infer the potential lineage differentiation trajectory based on the iCellR object.

### Identification of DEGs, Pathway Enrichment, Network Construction, and Molecular Complex Detection

DEG analysis was carried out in iCellR. We used the iCellR function run.diff.exp to identify DEGs between *Ezh2^fl/fl^* and *Ezh2^ΔNK^* cells within each stage. DEGs with padj < 0.05 were taken for further analysis. Identification of pathway and network enrichment and the MCODE algorithm were executed using Metascape (26) (http://metascape.org/). Accordingly, p-values were calculated based on the accumulative hypergeometric distribution, and q-values were calculated using the Banjamini–Hochberg procedure to account for multiple comparisons. Kappa scores were used as the similarity metric when performing hierarchical clustering on the enriched terms, and subtrees with a similarity > 0.3 were considered a cluster.

### Gene Motif Analysis

The putative promoter sequence was obtained from mm10 of the UCSC genome and then submitted to the Multiple Expectation Maximization for Motif Elicitation (MEME) *de novo* motif detection tool (meme mocular_complex.txt -dna -oc. -nostatus -time 14400 -mod zoops -nmotifs 10 -minw 6 -maxw 50 -objfun classic -revcomp -markov_order 0) and the Tool for Motif to Motif comparison (TOMTOM) (tomtom -no-ssc -oc. -verbosity 1 -min-overlap 5 -mi 1 -dist pearson -evalue -thresh 10.0 -time 300 query_motifs db/JASPAR/JASPAR2018_CORE_vertebrates_nonredundant.memedb/MOUSE/uniprobe_mouse.meme) for comparison with known motifs.

### Coexpression Network Construction

The WGCNA package of R software was applied to uncover correlations among genes. The gene expression matrix for each condition was extracted from iCellR. The power of β was set with the pickSoftThreshold function. The following analysis proceeded according to the official document provided by Dr. Jeremy Miller (https://horvath.genetics.ucla.edu/html/CoexpressionNetwork/JMiller/).

## Results

### NK Cells Were Separated Into Six Developmental Stages

**Figure 1:**
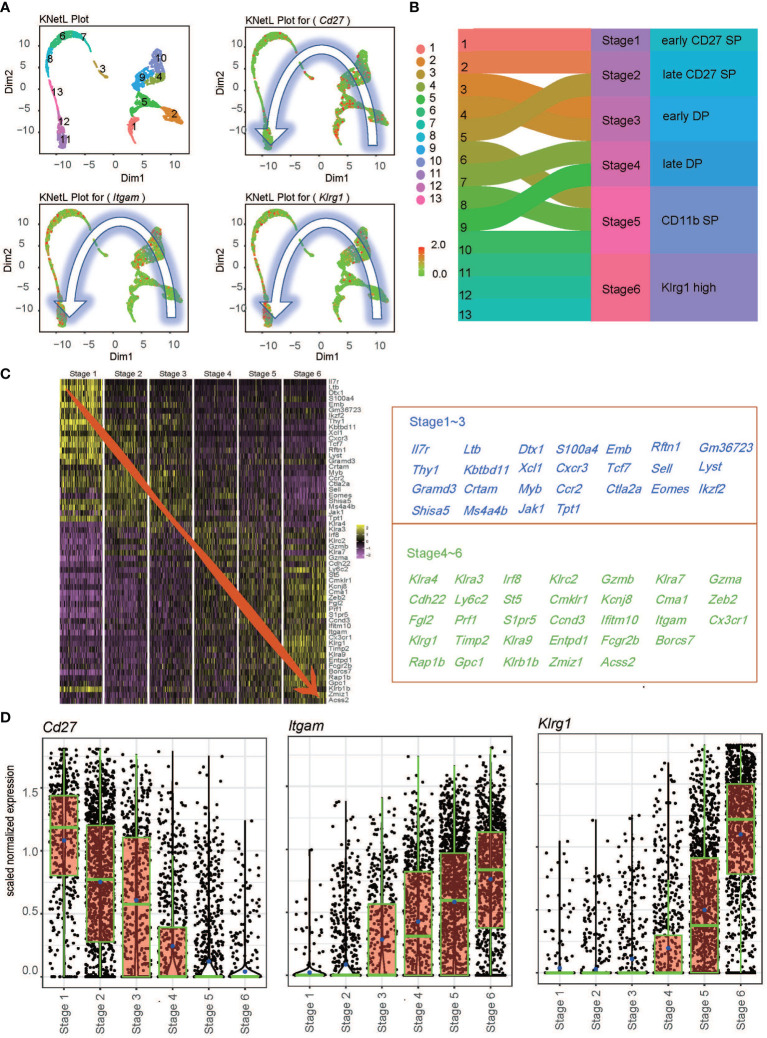
Transcriptional levels of conventional surface markers across the single-cell transcriptional profile. **(A)** Expression of Cd27, Itgam, and Klrg1 across 13 clusters defined by KnetL. The color key indicates iCellR imputed gene expression values. **(B)** Merging strategy based on the expression of Cd27, Itgam, and Klrg1 across 13 clusters. **(C)** Heatmap of the top 10 marker genes within the six stages (left) and gene list (right). **(D)** Boxplots demonstrate the expression of Cd27 and Klrg1 in each newly merged NK stage.

Our previous research showed that Ezh2 is a negative regulator of NK cell differentiation and function (17). However, the role of Ezh2 in regulating NK cell maturation remains unclear. To determine the role of Ezh2 in NK cell maturation, we sorted NK cells from the spleens of *Ezh2^fl/fl^* and *Ezh2^ΔNK^* mice to study the developmental heterogeneity of NK cells at the single-cell level using the BD Rhapsody single-cell gene expression system. A mean of 1,446 genes per cell among WT NK cells and 1,333 among *Ezh2* ^-/-^ NK cells were detected from 5,168 cells (2,584 in each condition). The KNN-based network graph drawing layout (KNetL) analysis showed higher resolution in unbiased clustering than t-distributed stochastic neighbor embedding (t-SNE) or uniform manifold approximation and projection (UMAP) analyses (**Figure S1**). KNteL analysis showed 13 distinct clusters from both mouse strains based on transcript signatures of WT and *Ezh2^-/-^* NK cells (**Figure 1** and **Figure S2**). The distributions of *Cd27*, *Itgam*, and *Klrg1* followed a counterclockwise pattern in the KNetL plot. Each cluster was independently compared to the ImmGen Ultra Low Input (ULI) RNA-seq dataset, which contained a total of 157 cell types, using a hypergeometric test. The top 10 predicted cell type for each cluster was then exhibited (**Figure S3**). All clusters were predicted to be likely NK cells and retained for further analysis. We found that Cluster 1 showed the highest *Cd27* expression and little *Itgam* expression; thus, this cluster was considered indicative of stage 1 (early CD27 SP NK cells). CD11b expression was initiated in Cluster 2 and Cluster 5 and then defined as corresponding to stage 2 (late CD27 SP NK cells). Stage 3 (early DP NK cells) and stage 4 (late DP NK cells) NK cells were identified based on the expression densities of *Itgam* and *Cd27* (**Figure S4**). Initiation of *Klrg1* expression occurred in stage 4. *Cd27* expression was mostly lost since stage 5 (CD11b SP NK cells), which consisted of Clusters 6, 8, and 10. Clusters within stage 5 undergo a process of diminishing *Cd27* expression, increasing *Itgam* expression, and steady but relatively moderate expression of *Klrg1* in the order of Cluster 10 → Cluster 6 → Cluster 8 (**Figure S4**). Clusters 11, 12, and 13 corresponded to stage 6 terminal CD27^-^CD11b^high^Klrg1^high^ NK cells (Klrg1 high NK) (**Figure 1B** and **Figure S4**). We identified 57 marker genes across stages, and these genes separated the stages into two tendencies (**Figure 1C**). Stages 1–3 demonstrated higher transcriptional levels of *Ltb, Emb, Thy1, Xcl1, Tcf7, Cxcr3, Ccr2*, and *Ctla2a*, while stages 3–6 showed augmented levels of *Ly6c2, Cma1, Gzma, Itgam, Klrg1, Rap1b*, and *Klra9*. (**Figure 1C**). Of note, we found that initiation of *Klrg1* transcription might be a late molecular event for *Itgam* (**Figure 1D**). In conclusion, we merged unbiased clusters into six stages—early CD27 SP, late CD 27 SP, early DP, late DP, CD11b SP, and Klrg1 high—based on the transcriptional levels of the conventional surface markers of NK cell development.

### Differential Cytotoxic Functions in *Ezh2*-Deficient NK Cells Were Intrinsically Associated With Alteration in the Developmental Process

**Figure 2:**
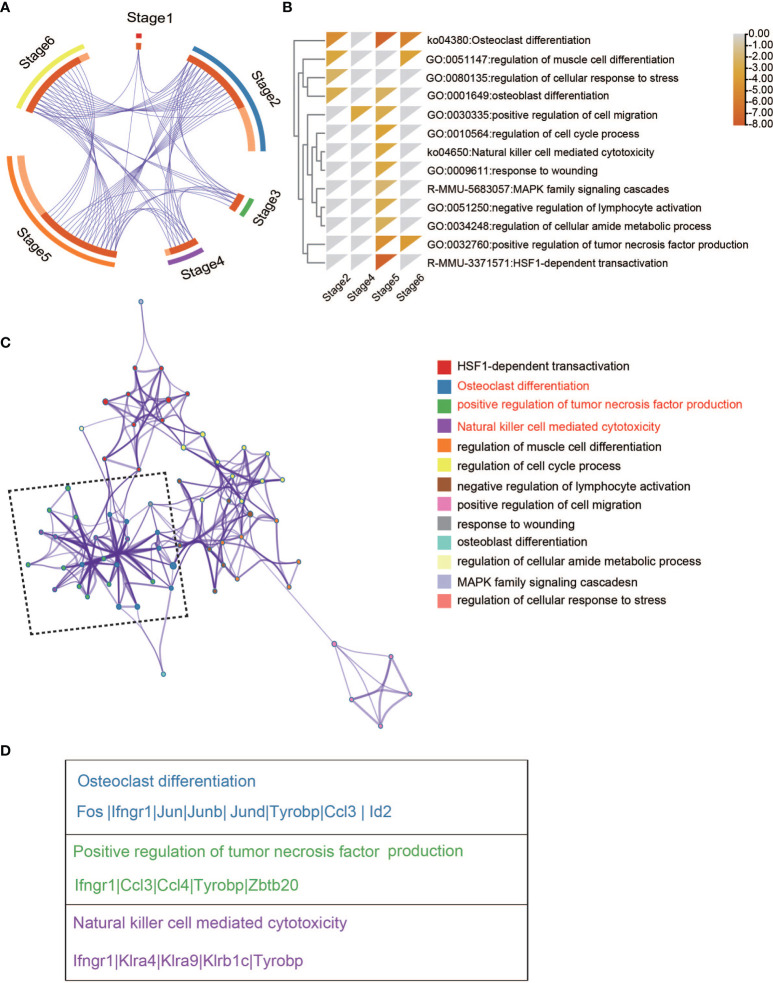
Enriched pathway network of DEGs between WT and Ezh2-/-NK cells. **(A)** Overlap between gene lists, where purple curves link identical genes. **(B)** Heatmap of enriched terms across input gene lists colored by p-values. **(C)** Network of enriched terms colored by cluster ID, where nodes sharing the same cluster ID are typically close to each other. **(D)** Gene lists of indicated pathways and processes.

To understand the differential gene expression pattern between WT and *Ezh2^-/-^* NK cells across six stages, we first determined the DEGs in two conditions within each stage (**Table S1**). A meta-analysis workflow (27) was used to combine DEGs between WT and *Ezh2*-deficient NK cells (**Figure 2A**). Interestingly, stage 2 and stage 5/6 shared many genes, implying that transcriptional alterations in stage 2 may have a cumulative effect in later stages. DEGs of CD11b SP stage 5 were enriched for terms related to natural killer cell-mediated cytotoxicity (ko34650) (**Figure 2B**). To further understand the biological processes that may influence NK cell cytotoxic function, a subset of enriched terms was selected and rendered as a network plot, where terms with a similarity > 0.3 were connected by edges. We selected the terms with the best p-values from each of the 20 clusters under a constraint of no more than 15 terms per cluster and no more than 250 terms in total. The enrichment network showed that the natural killer cell-mediated cytotoxicity term shared a close relationship with terms related to positive regulation of tumor necrosis factor production and osteoclast differentiation (**Figure 2C**). Of note, genes enriched in the osteoclast differentiation term, such as *Id2* (28), *Ccl3* (24), and *Tyrobp* (29), have been reported to also promote NK cell development (**Figure 2D**). These results suggested that the differential cytotoxic functions of NK cells may share intrinsic connections with an altered development process.

### Maturation Trajectory of *Ezh2* ^-/-^ NK Cells Arresting in CD11b SP Stage 5

**Figure 3:**
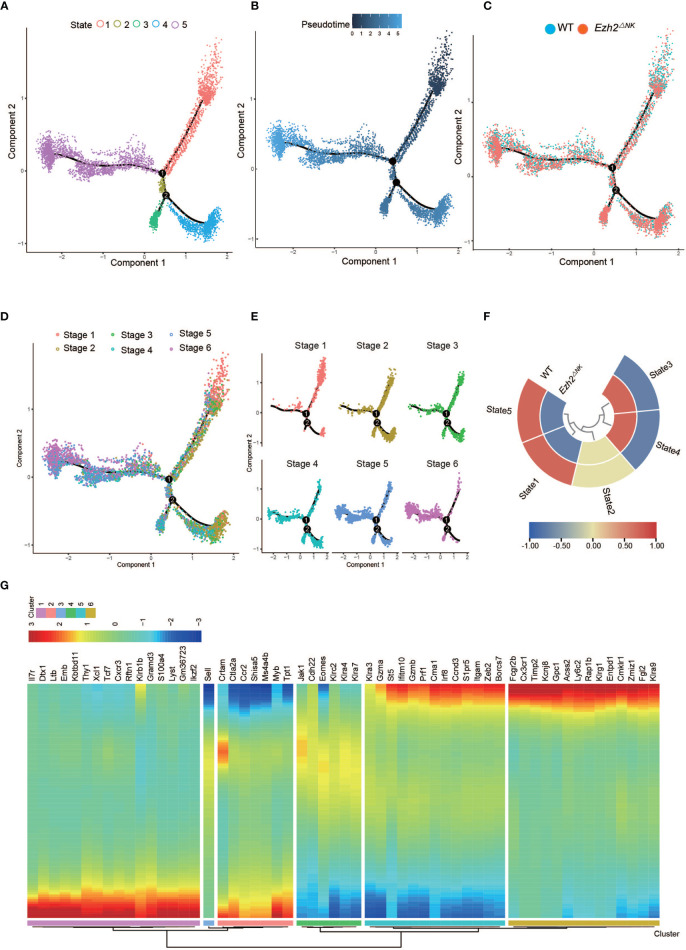
The heterogeneity of relative maturity between WT and Ezh2^ΔNK^ mice. **(A)** The relative maturity along the developmental trajectory is displayed across pseudotime. **(B)** Distribution of five developmental states defined by monocle along the pseudotime trajectory. **(C)** Distribution of all conditions along the pseudotime trajectory. **(D)** Distribution of all NK stages along the pseudotime trajectory. **(E)** Heatmap of normalized cell numbers of each NK stage in the pseudotime trajectory states. **(F)** The compositions of WT and Ezh2^-/-^ NK cells in five states. **(G)** Heatmap showing clustering genes by pseudotemporal expression pattern.

To explore the differential development process between WT NK cells and *Ezh2*-deficient NK cells, we used Monocle2 pseudotime analysis to simulate the maturation trajectory based on the marker gene in **Figure 1**. Cells from each of the six stages were assigned to the pseudotime trajectory with five states and two trajectories (**Figure 3A**). The pseudotime progression indicated the maturation order (**Figure 3B**). WT NK cells took advantage in trajectory 1, and *Ezh2^-/-^* NK cells dominated trajectory 2 (**Figures 3C, F**). The root point of trajectory 1 (up, state 1,5) is obvious upright as stage 1 mostly resided here. Based on this root point, the pseudotime dictated that the up-left side of trajectory 1 corresponded to the most mature population, which was dominated by stage 6. The cells in stage 2 to stage 5 spread across the trajectory from right to left. Trajectory 1 shows a classical process of NK cell maturation (**Figures 3D, E**). However, trajectory 2 (down, state 3, 4) seemed to skip stage 1 in the down-right. Interestingly, while stages 2, 3, 4, and 5 also spread from down-right state 4 to down-left state 3 in trajectory 2, stage 6 (Klrg1 high) was likely to be aborted in down-left side state 3 since stage 5 (CD11b SP) dominated this root (**Figures 3D, E**).

Differential cytotoxic functions in *Ezh2*-deficient NK cells shared intrinsic connections with altered development processes (**Figure 2**). Thus, pseudotime-dependent genes were clustered by pseudotemporal expression patterns. *Itgam* and *Klrg1* were separately grouped into Cluster 5 and Cluster 6, indicating that they followed differential kinetic trends. Of note, *Gzma, Gzmb, Prf1*, and *Irf8* were clustered with *Itgam* (**Figure 2**), which is associated with NK cell antitumor function (18–20). *Fcgr2b, Fgl2, Cx3cr1*, and *Klra9* (Ly49C) were grouped into Cluster 6 with *Klrg1* (**Figure 2**). The expression of Fcgr2b, Fgl2, and Ly49C is known to impair the cytotoxic function of NK cells (21–23). Cx3cr1^+^ NK cells have been reported to show less proliferation potential than Cx3cr1^-^ NK cells (30). These results indicate that CD11b SP NK cells may gain a cytotoxic capacity exceeding that of Klrg1-high NK cells. Therefore, these results suggested that deletion of *Ezh2* may restrain the terminal Klrg1-high NK cell pool and lead to enhanced cytotoxicity.

### *Ezh2*-Deficient NK Cells Exhibited a Lower Transcriptional Level of Members of the AP-1 Family

**Figure 4:**
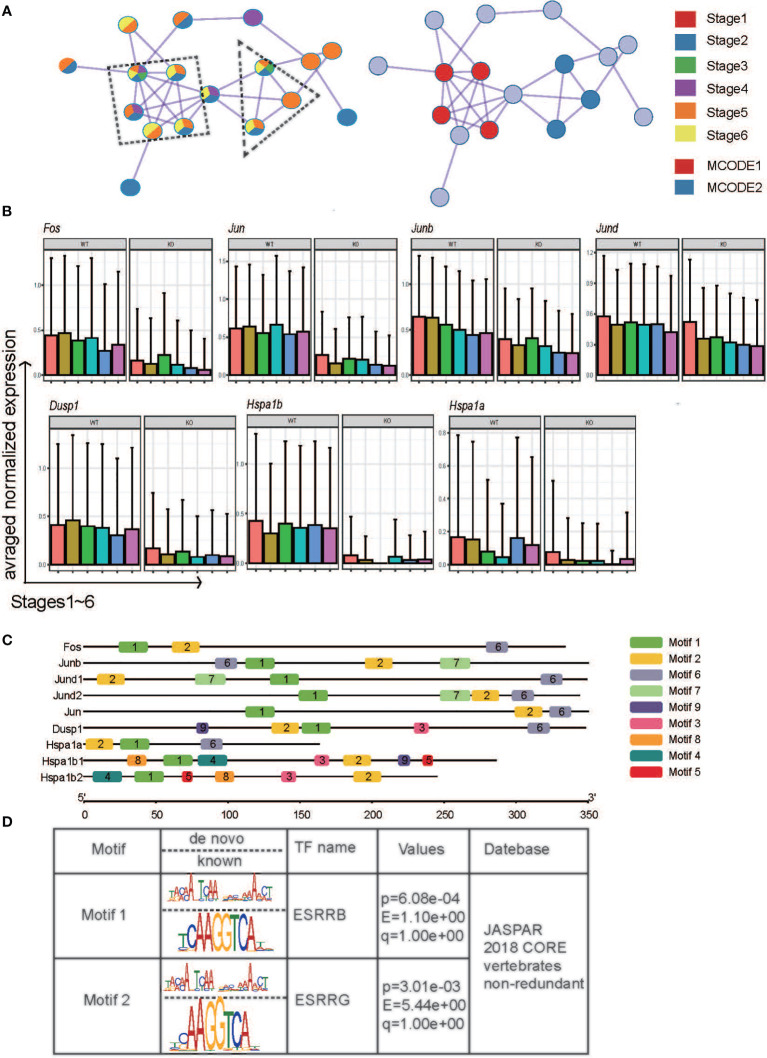
The potential mechanism by which Ezh2 regulates the expression of the indicated genes. **(A)** Components identified by the MCODE algorithm of Metascape analysis. **(B)** Boxplot of the indicated genes at different stages under the two conditions. **(C)** Distribution of PWM generated by the meme suite across promoters of the indicated genes. **(D)** The transcription factors predicted by TOMTOM.

Interestingly, the frequencies of Klrg1^+^ NK cells were the same in *Ezh2^ΔNK^* mice compared with WT mice by flow cytometry (data not shown), although robust expansion of the CD27^-^CD11b^+^ NK pool was observed. To further explain this phenomenon, we identified a densely connected network of protein–protein interactions in DEGs using MCODE. Two key modes of densely connected network components were identified. MCODE1 is composed of *Fos, Junb, Jund*, and *Jun* (AP-1 superfamily), while *Dusp1, Hspa1a*, and *Hspa1b* (HSF-1 family) constitute MCODE2 (**Figure 4A**). The expression patterns of the indicated genes across stages between conditions were described. The genes were mostly downregulated in *Ezh2^-/-^* NK cells across six stages compared with WT NK cells (**Figure 4B**), which seems to contradict reports indicating that Ezh2 is a negative regulator of chromatin activation (31). To further explore the underlying mechanism, the promoter sequences of these genes were obtained from the UCSC Genome Browser (**Table S2**). Analysis of conserved motif distributions was performed using the motif analysis online program MEME (32). Motif 1 and Motif 2 were distributed across promoters of all genes (**Figure 4C**). Submission of the position weight matrix of motifs to TOMTOM (33) revealed that Motif 1 recognizes DNA and is most similar to the estrogen-related receptor family binding motif (**Figure 4D**).

We also performed the WGCNA. The most preserved module between the two conditions is turquoise (**Figure S5A** and **Table S3**). As expected, most of the regulators of NK cell maturation and function were in turquoise (**Table S4**), such as Il2rb, Cd27, Ifng, Nfkb1, Smad3, Tcf7, Bcl2, Foxo1, Gata3, Stat2, and Stat4 (5). Venn diagram showed the overlap between the Turquoise module genes and DEGs. Interestingly, members of AP-1 family Junb, Jund, and Fos belong to both DEGs and the turquoise module (**Figure S5B**). This agrees with our conclusion that the AP-1 family plays an important role in regulating the maturation and function of Ezh2-deficiency NK cells. Overall, our results suggested that deletion of Ezh2 may alter the NK maturation trajectory and enhance cytotoxic function by attenuating the AP-1 superfamily.

## Discussion

The role of NK cells in the development and function of epigenetic modifications remains poorly understood. Specifically, histone modification during NK cell ontology is only partially defined. Li et al. found that the histone modification state has a profound impact on NK cell activation (34). During acute NK cell activation, the epigenetic and transcriptional profiles are altered, and p300 enhancer modifications are also involved in high-level transcriptional induction of T-bet and STATs to facilitate the rapid immune response (35). Kdm5a, an H3K4me3 demethylase, is a positive modifier of NK cell activation and is recruited to the SOCS1 promoter by p50 to maintain a repressive chromatin configuration, which promotes NK cell production of interferon-γ (IFN-γ) (36). Adam et al. identified histone H3K27 demethylases as key regulators of cytokine production using small-molecule inhibitors of chromatin methylation and acetylation screens in human NK cells, and GSK-J4 is an H3K27 demethylase inhibitor that can increase global levels of the repressive H3K27me3 chromatin marker and lead to suppression of the anti-inflammatory response (37). Interestingly, in our previous research, we found that Ezh2 deletion in *Vav-iCre* mice led to alterations in differentiation and enhanced function following Vav expression (17). Here, we found that loss of Ezh2 is required to constrain terminal and functionally impaired Klrg1-high NK cells.

ScRNA-seq is a powerful technique allowing unbiased identification of cellular diversity and heterogeneous transcriptional signatures within one cell population (38, 39). Our results provide a transcriptional landscape of individual NK cells from the mouse spleen and a clustered subpopulation of NK cells based on the distribution of surface markers. We identified six stages based on the transcriptional levels of *Cd27*, *Itgam*, and *Klrg1*: early CD27 SP stage 1, late CD27 SP stage 2, early DP stage 3, late DP stage 4, CD11b SP stage 5, and Klrg1 high stage 6 (**Figure 1B**). Trends in marker gene expression were basically consistent with those reported in previous research by Adeline Crinier et al. (40), as shown in **Figure 1C**. We noticed that differential NK cytotoxicity was intrinsically connected with altered developmental processes (**Figures 2C, D**). Furthermore, we found that Ezh2-deficient NK cells follow a maturation trajectory toward arrest before Klrg1 high stage 6 (**Figure 3C**). NK cells have been reported to acquire the terminal marker Klrg1 and become less responsive to proliferative signals following a decrease in the turnover rate and homeostatic expansion *in vivo* (8). Several studies have shown that Klrg1 inhibits NK cell activation (9) and cytotoxicity (10). Consistent with this, we found that *Klrg1* was clustered with genes that are known to impair NK cell proliferation and cytotoxicity by a pseudotemporal expression pattern (**Figure 3G**). Thus, we speculated that enhanced cytotoxicity is due to a distinct maturation trajectory in *Ezh2*-deficient NK cells.

The AP-1 family is a pioneer transcription factor (TF) family with an important role in establishing new cell fate competence by granting long-term chromatin access to nonpioneer factors and is also a crucial determinant of cell identity through opening and licensing of the enhancer landscape (41, 42). In particular, AP-1 TFs recruit the SWI/SNF (BAF) complex to enhancers to establish accessible chromatin in fibroblasts (43). Jeong SM et al. found that the SWI/SNF chromatin-remodeling complex modulates peripheral T cell activation and proliferation by controlling AP-1 expression. Our previous research confirmed that tumor formation following SNF5 loss leads to elevated expression of EZH2 (44). Stefaniewe et al. found that Fra-2/AP-1 is regulated through lysine methylation by Ezh2 in terminal epidermal differentiation (45). Thus, we speculate that the AP-1 family plays an important role in EZH2-mediated chromatin remodeling.

Recently, the role of the AP-1 family in the immune response has also been explored (46, 47). BATF constitutes an important subset of the AP-1 superfamily (48) and forms a heterodimer complex containing AP-1 and JUN proteins to promote transcriptional activation (47). For example, Miller et al. found that the numbers and function of Klrg1-high ILC2 cells are impaired in the IL-25-mediated helminth clearance model in BATF-deficient mice (11). Consistently, as shown in **Figure 4A**, we found differential expression patterns of the AP-1 superfamily between the two mouse strains by MCODE. We further confirmed that AP-1 superfamily members were downregulated in Ezh2^-/-^ NK cells compared to WT NK cells at each stage (**Figure 4B**). In our WGCNA, the AP-1 family was found to contain modules with many important regulators of NK cell maturation and function. Thus, we speculate that the AP-1 family plays an important role in Ezh2-mediated regulation of NK cell maturation and cytotoxicity. Lau et al. found that *Klrg1* was within regions with the most variable chromatin accessibility during MCMV infection. Of note, members of the AP-1 transcription factor family, especially Junb and Jund, are found in regions that become more accessible to memory NK cells than to naïve NK cells (49). Our results supported that the members of AP-1 superfamily skew the Klrg1^+^ NK population.

A nonclassical regulatory mechanism of Ezh2 exists in cancer cells. For example, Ezh2 can activate the transcription of downstream target genes through methylation of nonhistone proteins (50, 51) or directly form transcription complexes with other factors independent of PRC2 (15, 16, 52). Jiao L et al. reported that Ezh2 contains a hidden, partially disordered transactivation domain (TAD), which directly binds to the transcriptional coactivator p300 and activates gene expression (14). Specifically, Shi B et al. reported that Ezh2 physically interacts directly with estrogen receptor alpha and functionally enhances gene transactivation (15). We found that motifs enriched in promoters of the indicated genes are most likely to be the binding site of ERRs, which are structurally similar to the ER (53, 54). Our study implied that Ezh2 may act as a transcriptional activator in the regulation of AP-1 superfamily gene expression by interacting with ERRs. In conclusion, our results implied a novel role for the Ezh2-AP-1-Klgr1 axis in altering the NK cell maturation trajectory and NK cell-mediated cytotoxicity.

## Data Availability Statement

The sequencing data has been deposited in the NCBI GEO database (https://www.ncbi.nlm.nih.gov/geo/; accession number: GSE179186).

## Ethics Statement

The animal study was reviewed and approved by Capital Medical University.

## Author Contributions

XW conceived and supervised the study as well as revised the manuscript. MY and ZS designed the research, exported the figures, wrote the first draft of the manuscript, and performed the verification experiment.XH helped in performing the experiments. All authors contributed to the article and approved the submitted version.

## Funding

This work was supported by grants from the Ministry of Science and Technology of People’s Republic of China (grant# 2014CB910100 to XW), the Support Project of High-level Teachers in Beijing Municipal Universities in the Period of 13th Five-year Plan (IDHT20190510 to XW), and the National Natural Science Foundation of China (81972652 to XW).

## Conflict of Interest

The authors declare that the research was conducted in the absence of any commercial or financial relationships that could be construed as a potential conflict of interest.

## Publisher’s Note

All claims expressed in this article are solely those of the authors and do not necessarily represent those of their affiliated organizations, or those of the publisher, the editors and the reviewers. Any product that may be evaluated in this article, or claim that may be made by its manufacturer, is not guaranteed or endorsed by the publisher.

## Supplementary Material

Supplementary Figure 1 PCA, UMAP, tSNE and KnetL plots of clusters.

Supplementary Figure 2 Percentages of NK cells from the indicated conditions across thirteen clusters.

Supplementary Figure 3 Top 10 cell types identified using the ImmGen Ultra Low Input (ULI) RNA-seq dataset against the genes within each cluster by a hypergeometric test.

Supplementary Figure 4 Boxplots of Cd27, Itgam, and Klrg1 across the original 13 clusters in **(A)**, WT mice and Ezh2^ΔNK^ mice or across 6 newly merged stages in WT mice and Ezh2^ΔNK^ mice.

Supplementary Figure 5
**(A)** Hierarchical cluster tree showing coexpression modules of WT NK cells (left) identified by WGCNA and their preservation in Ezh2-deficient NK cells (right). **(B)** Venn diagram showing the overlap between the Turquoise module genes and DEGs.
