# Supplementary figures and images for: Single-Cell Sequencing Reveals the Novel Role of Ezh2 in NK Cell Maturation and Function
Source: Front Immunol. 2021 Oct 26;12:724276. doi: 10.3389/fimmu.2021.724276 (PMC8576367; doi:10.3389/fimmu.2021.724276)

Supplemental Figure 1

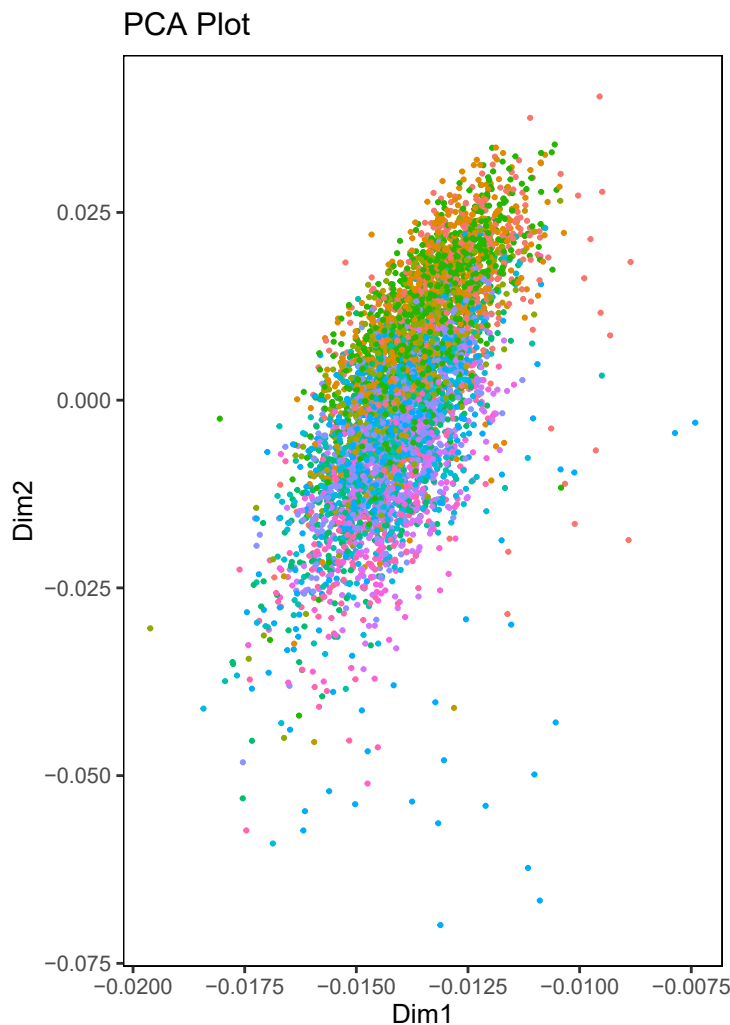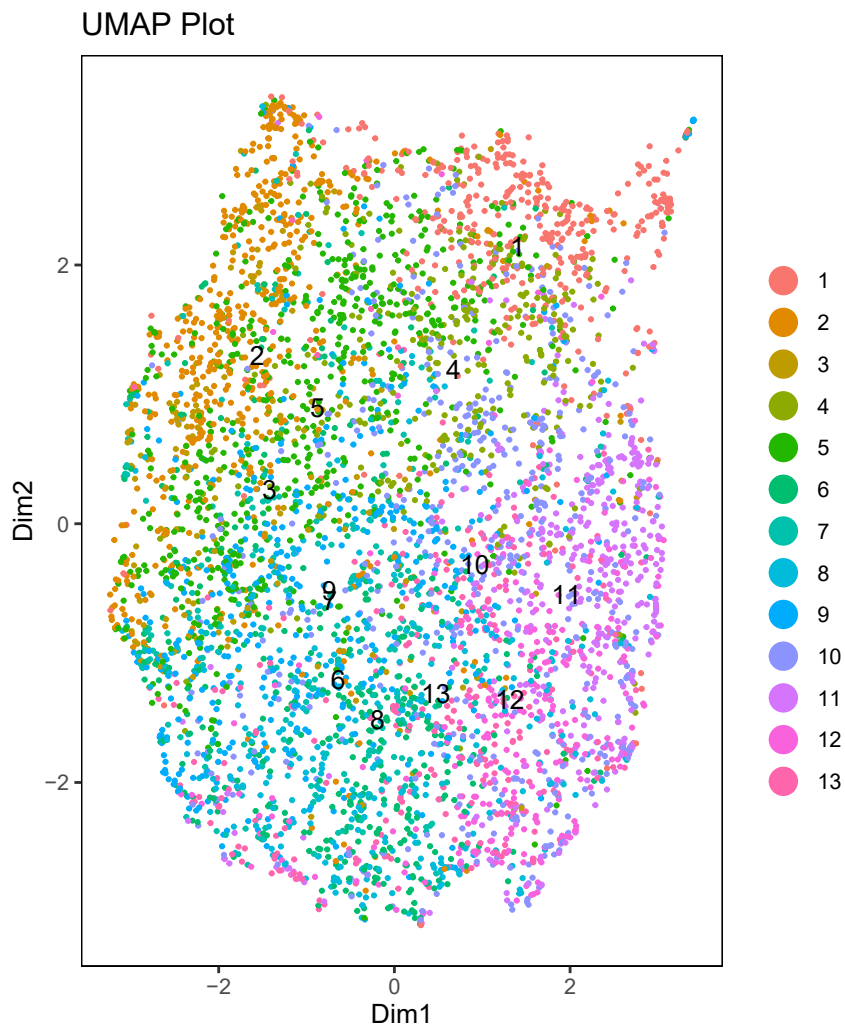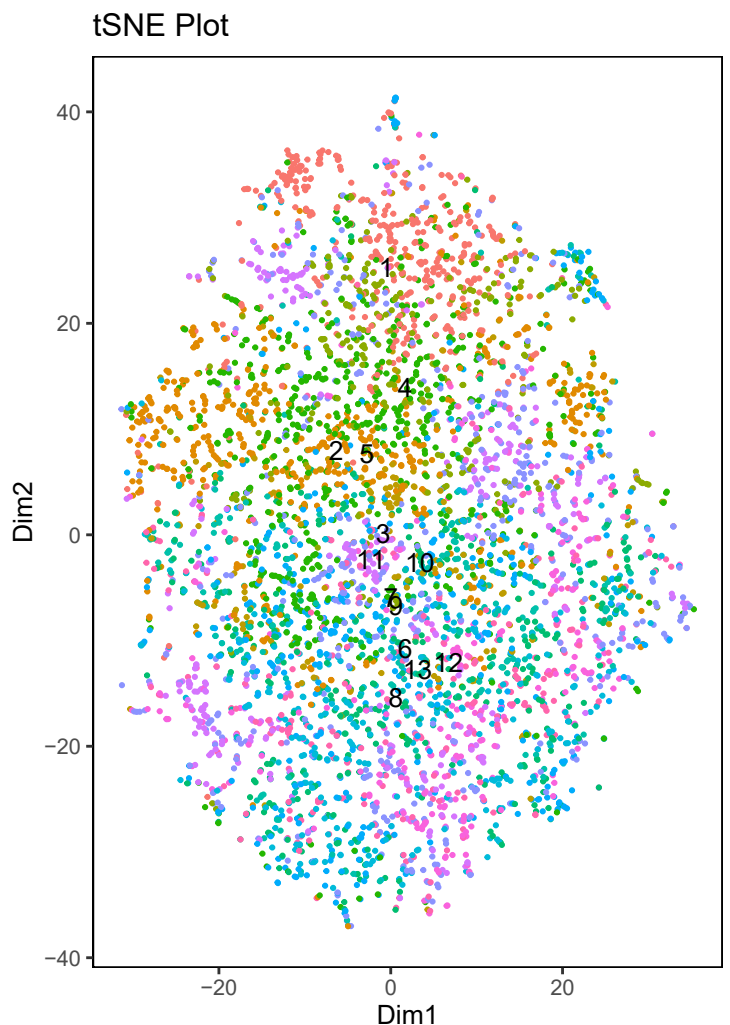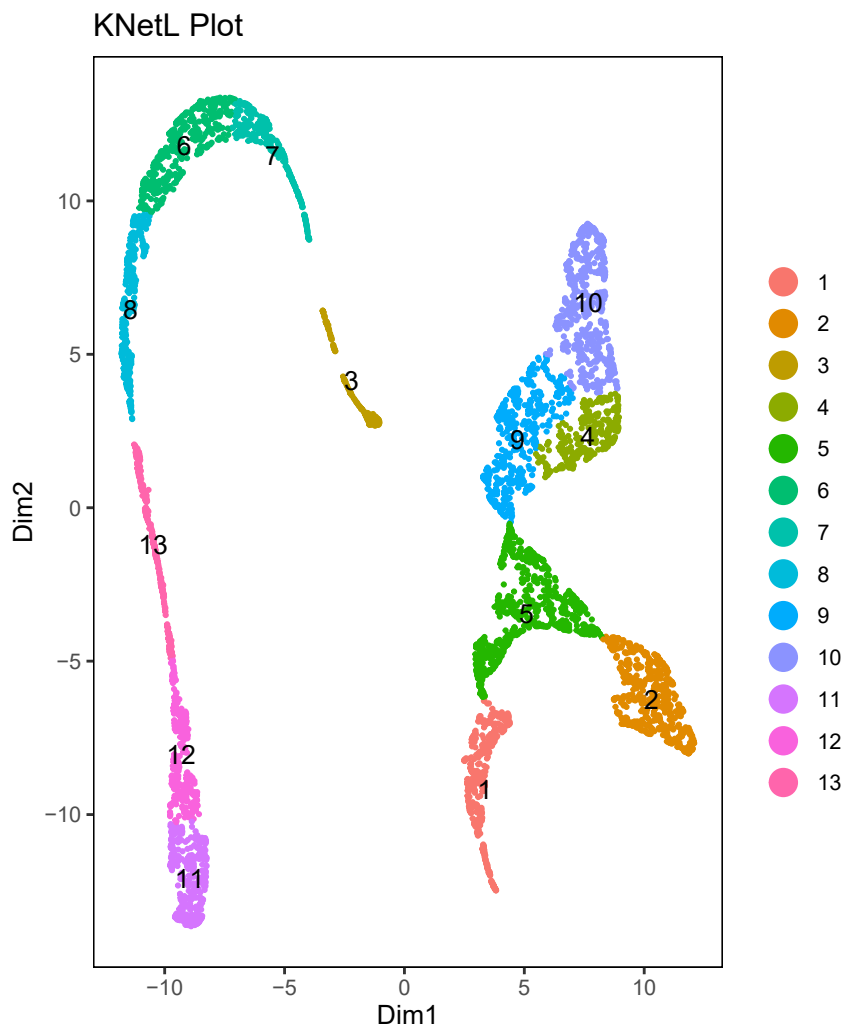

Supplement: Supplementary Figure 1 — PCA, UMAP, tSNE and KnetL plots of clusters. [file DataSheet_1.pdf]

Supplemental Figure 2

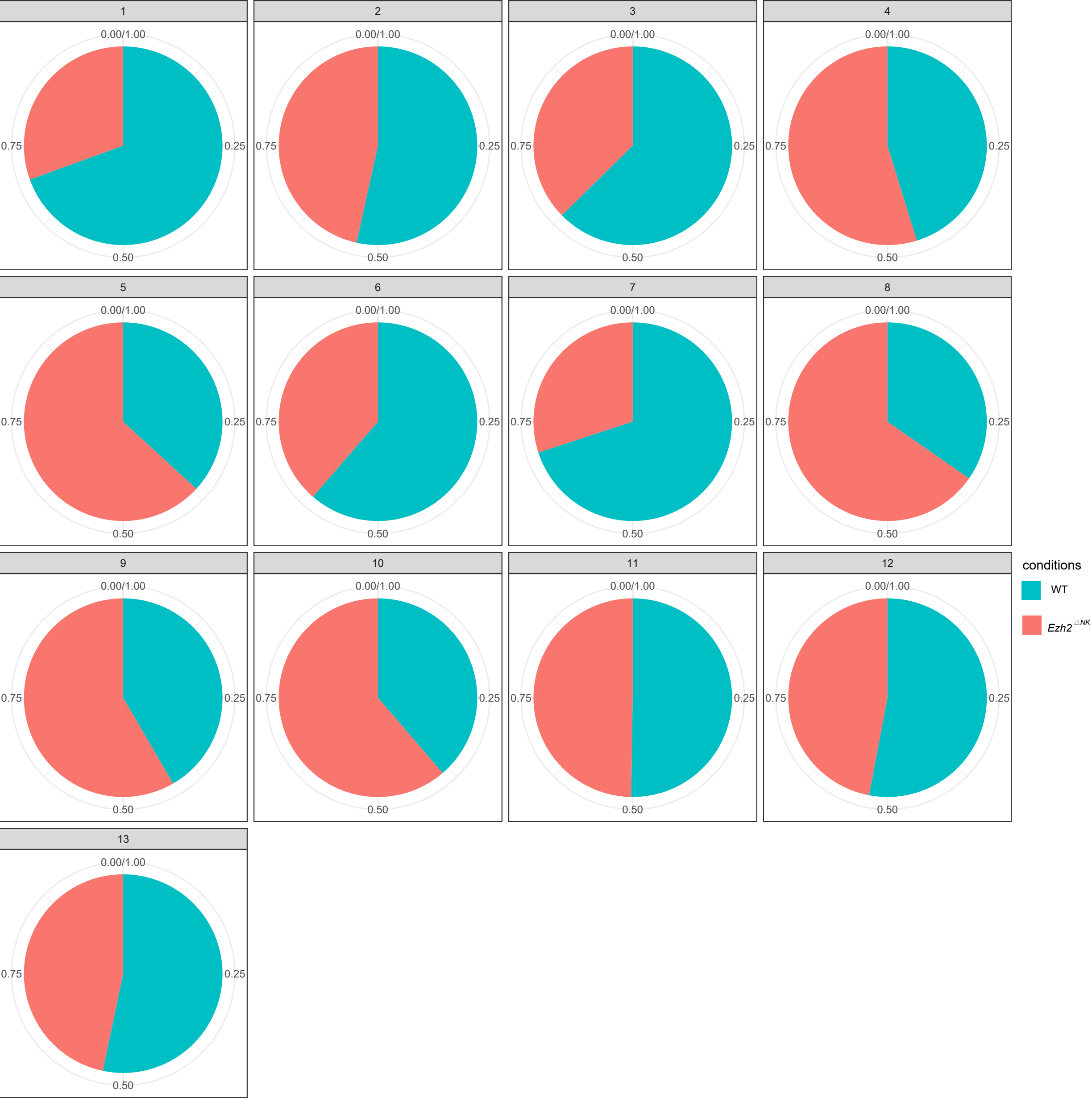

percentage

Supplement: Supplementary Figure 2 — Percentages of NK cells from the indicated conditions across thirteen clusters. [file DataSheet_2.pdf]

# Supplemental Figure 3

Top 10 out of 157 (ImmGen ultra-low-input RNA-seq)

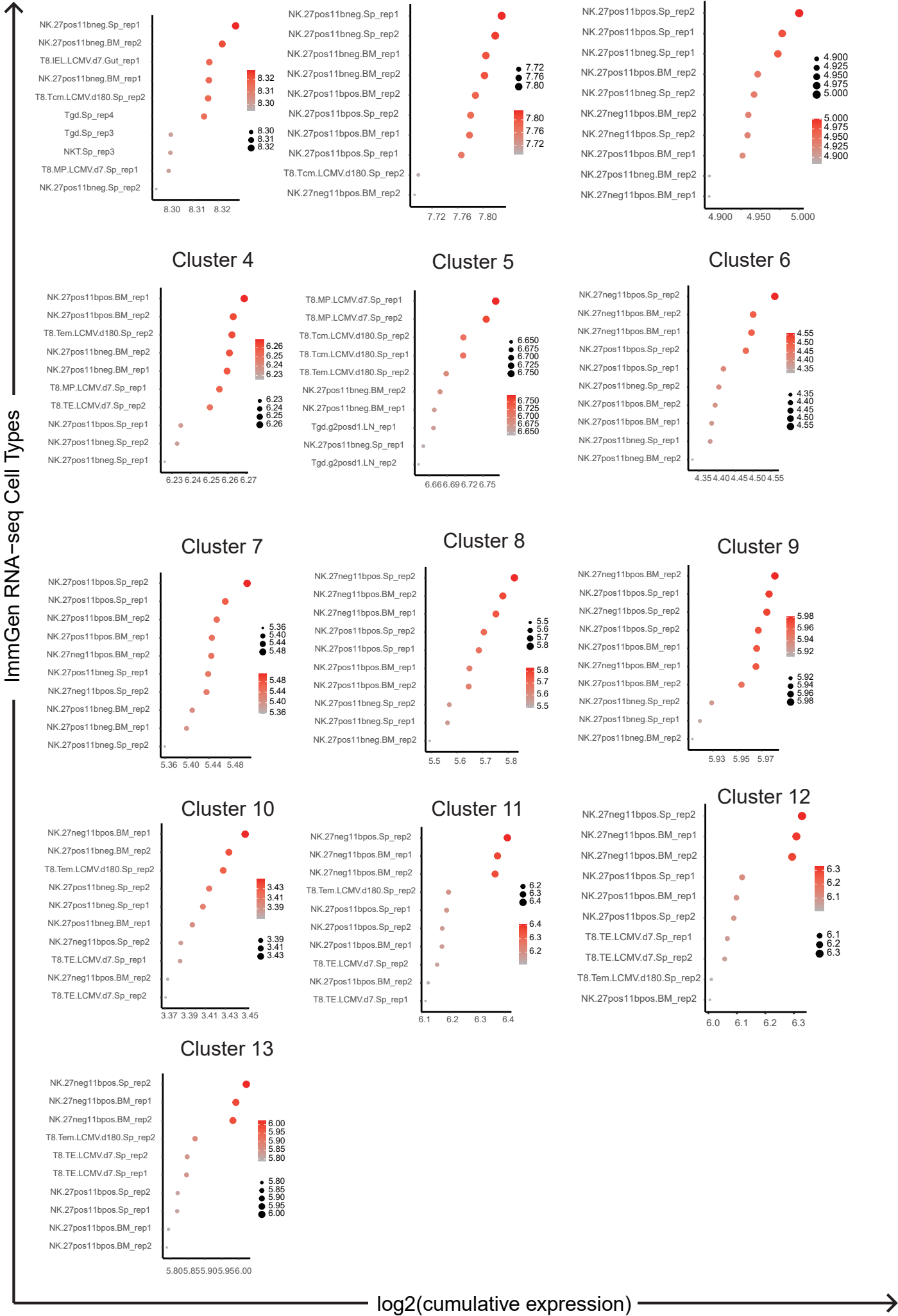

Supplement: Supplementary Figure 3 — Top 10 cell types identified using the ImmGen Ultra Low Input (ULI) RNA-seq dataset against the genes within each cluster by a hypergeometric test. [file DataSheet_3.pdf]

# Supplemental Figure 4

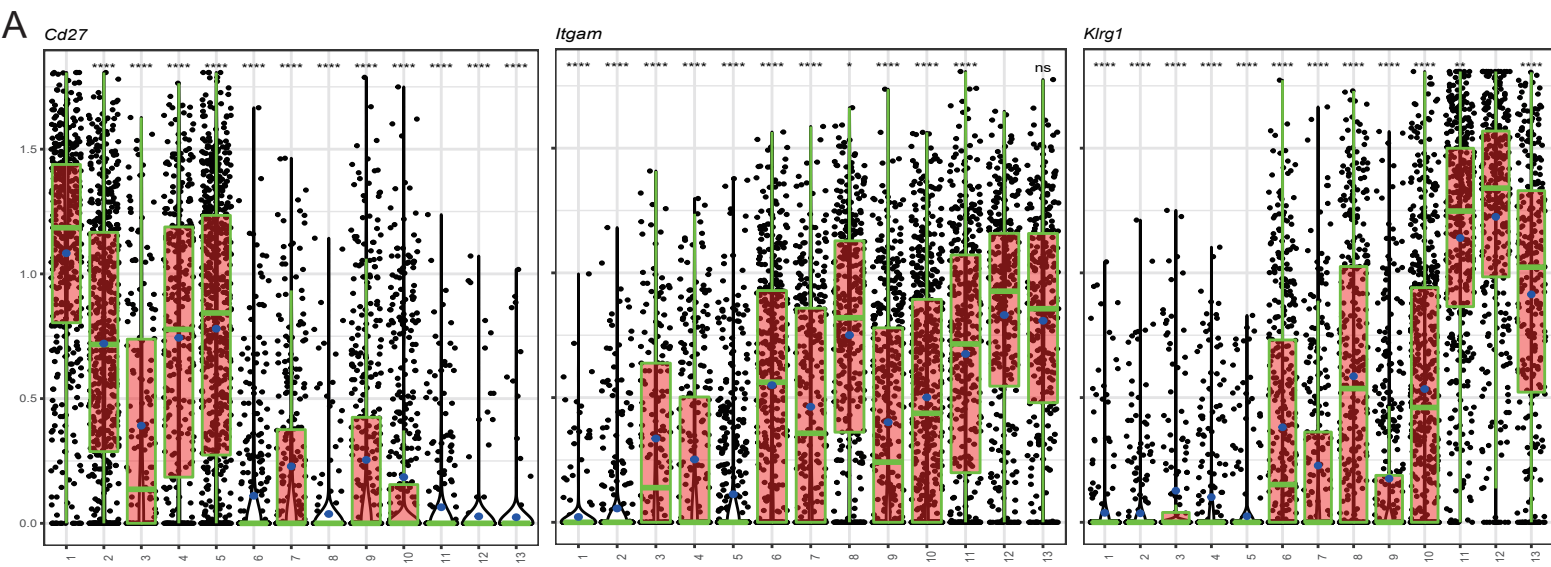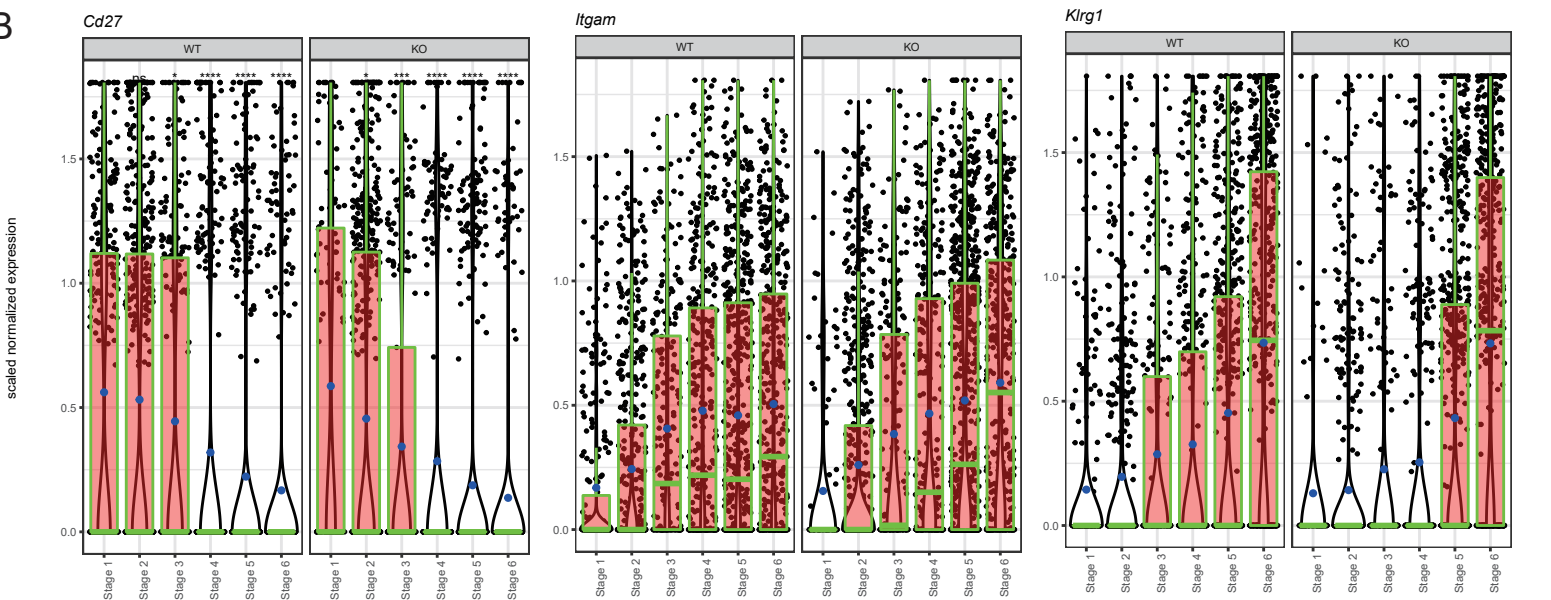

Supplement: Supplementary Figure 4 — Boxplots of Cd27, Itgam, and Klrg1 across the original 13 clusters in (A), WT mice and Ezh2ΔNK mice or across 6 newly merged stages in WT mice and Ezh2ΔNK mice. [file DataSheet_4.pdf]

Supplemental Figure 5

A

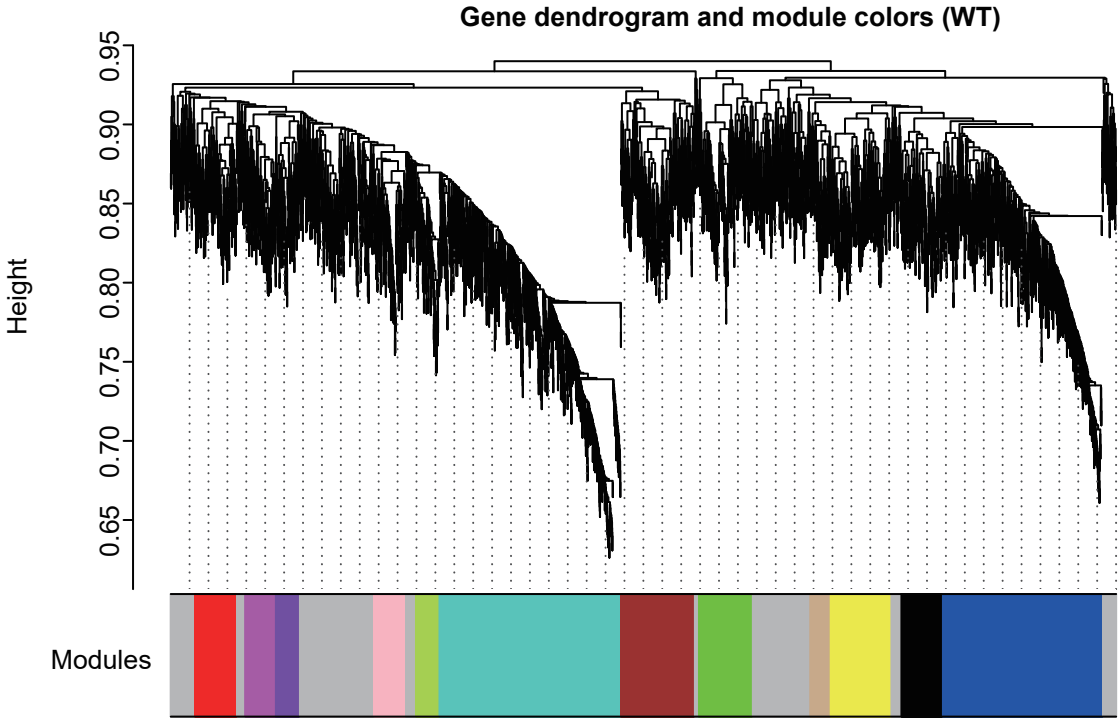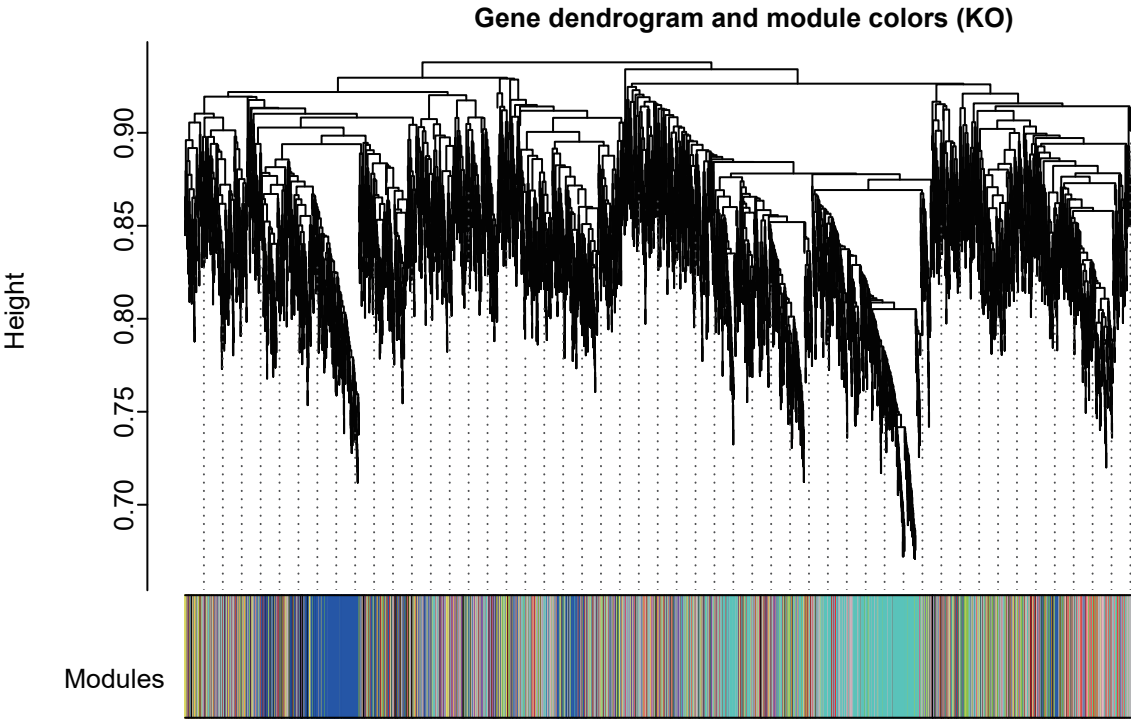

B

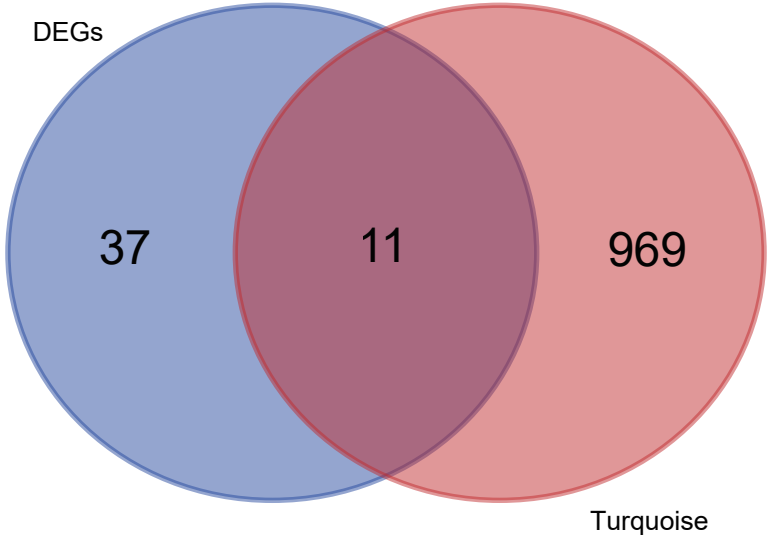

Supplement: Supplementary Figure 5 — (A) Hierarchical cluster tree showing coexpression modules of WT NK cells (left) identified by WGCNA and their preservation in Ezh2-deficient NK cells (right). (B) Venn diagram showing the overlap between the Turquoise module genes and DEGs. [file DataSheet_5.pdf]
